# Prolonged co-treatment with HGF sustains epithelial integrity and improves pharmacological rescue of Phe508del-CFTR

**DOI:** 10.1038/s41598-018-31514-2

**Published:** 2018-08-29

**Authors:** Ana M. Matos, Andreia Gomes-Duarte, Márcia Faria, Patrícia Barros, Peter Jordan, Margarida D. Amaral, Paulo Matos

**Affiliations:** 1 Department of Human Genetics, National Health Institute ‘Dr. Ricardo Jorge’, Av. Padre Cruz, 1649-016 Lisboa, Portugal; 2 University of Lisboa, Faculty of Sciences, BioISI – Biosystems & Integrative Sciences Institute, Campo Grande – C8, 1749-016 Lisboa, Portugal; 3 Serviço de Endocrinologia, Diabetes e Metabolismo, do CHLN – Hospital Santa Maria, Lisboa, Portugal

## Abstract

Cystic fibrosis (CF), the most common inherited disease in Caucasians, is caused by mutations in the CFTR chloride channel, the most frequent of which is Phe508del. Phe508del causes not only intracellular retention and premature degradation of the mutant CFTR protein, but also defective channel gating and decreased half-life when experimentally rescued to the plasma membrane (PM). Despite recent successes in the functional rescue of several CFTR mutations with small-molecule drugs, the folding-corrector/gating-potentiator drug combinations approved for Phe508del-CFTR homozygous patients have shown only modest benefit. Several factors have been shown to contribute to this outcome, including an unexpected intensification of corrector-rescued Phe508del-CFTR PM instability after persistent co-treatment with potentiator drugs. We have previously shown that acute co-treatment with hepatocyte growth factor (HGF) can significantly enhance the chemical correction of Phe508del-CFTR. HGF coaxes the anchoring of rescued channels to the actin cytoskeleton via induction of RAC1 GTPase signalling. Here, we demonstrate that a prolonged, 15-day HGF treatment also significantly improves the functional rescue of Phe508del-CFTR by the VX-809 corrector/VX-770 potentiator combination, in polarized bronchial epithelial monolayers. Importantly, we found that HGF treatment also prevented VX-770-mediated destabilization of rescued Phe508del-CFTR and enabled further potentiation of the rescued channels. Most strikingly, prolonged HGF treatment prevented previously unrecognized epithelial dedifferentiation effects of sustained exposure to VX-809. This was observed in epithelium-like monolayers from both lung and intestinal origin, representing the two systems most affected by adverse symptoms in patients treated with VX-809 or the VX-809/VX-770 combination. Taken together, our findings strongly suggest that co-administration of HGF with corrector/potentiator drugs could be beneficial for CF patients.

## Introduction

Mutations in the CFTR gene cause cystic fibrosis (CF), the most common inherited disease in Caucasians^1^. They can alter the synthesis, processing, function, and half-life of CFTR, the main chloride channel expressed apically at the plasma membrane (PM) of epithelial cells^2^. Respiratory failure, derived from severe airway obstruction, inflammation, and recurrent infections, is the most prevalent mortality cause in CF^2^. Although CF is still incurable (mean survival of ~40 years), the last decade brought remarkable progress towards personalized CF treatments, with the development of small-molecule drugs targeting frequent CFTR mutations^3,4^. The proof-of-principle was Ivacaftor (VX-770), a potentiator drug that relieved CF symptoms in patients bearing mutations (e.g., Gly551Asp) that impair CFTR channel activity (4–8% of patients)^5^. Soon after, Lumacaftor (VX-809), a pharmacological chaperone, in combination with VX-770, was approved by the Food and Drug Administration (FDA) and European Medicines Agency (EMA)^6^ for patients homozygous for the most common mutation – Phe508del (~40% of patients), which impairs CFTR folding and trafficking^7^. In phase II trials, the VX-809/VX-770 combination (at the higher administered doses) significantly improved the percentage predicted forced expiratory volume in one second (FEV_1_) by a mean of 6% in patients homozygous for Phe508del-CFTR, decreased sweat chloride concentration by ~10 mmol/L, and decreased pulmonary exacerbations in the treatment groups^8^. Data from subsequent phase III trials revealed improvements in predicted FEV_1_ ranging from 2.6–4.0% and a clear, 30–39% decrease in the rate of pulmonary exacerbations, significantly reducing hospitalization and the use of intravenous antibiotics in the treatment groups^9^. The VX-809/VX-770 drug combination has now been used in patients since 2015, and several subsequent studies of its long-term usage indicate that it does benefit CF patients, although several cases of off-target side-effects have been reported, the most frequent being respiratory and gastrointestinal manifestations^10–12^.

Despite the reported benefits, the results from these studies fell below initial expectations and experimental evidence emerged to, at least partially, explain the limited clinical improvements observed in patients. For instance, it was shown that persistent exposure to potentiator drugs, particularly VX-770, results in a dose-dependent reversal of VX-809-mediated CFTR correction in Phe508del-CFTR homozygous primary airway cell cultures^13,14^. This was due to destabilization and increased turnover of the rescued protein, resulting in its reduced functional expression at the cell surface. A posterior study, however, argued that at clinically relevant concentrations (below 1 μM), continuous exposure to VX-770 does not inhibit the rescue of Phe508del-CFTR by VX-809^15^. In addition, it was also observed that *Pseudomonas aeruginosa* reduces Phe508del-CFTR function in cells treated either with VX-809 alone or with the VX-809/VX-770 combination^16,17^. Since 85% of adult CF patients are colonized with *P. aeruginosa*, these data suggest that infection with these bacteria may also contribute to a reduction in the therapeutic efficacy of these drugs.

Importantly, we and others showed that the intrinsically reduced stability of rescued Phe508del-CFTR at the cells’ PM (less than 10% that of wt-CFTR)^18,19^ -due to its deficient anchoring to the actin cytoskeleton and targeting by the peripheral protein quality control - could be a major obstacle to its pharmacological correction^19–22^. However, we showed that acute treatment with hepatocyte growth factor (HGF) can increase Phe508del-CFTR stability and retention at the PM^20^. HGF acts via RAC1 GTPase to promote the PDZ-mediated interaction of Phe508del-CFTR with NHERF1 and ezrin adaptor proteins, favouring the channel’s anchoring to the actin cytoskeleton^20,21^. Notably, co-treatment with HGF enhanced over 3-fold the functional restoration efficacy of chemical correctors such as corr-4a (C4) and C3, by preventing internalization of rescued Phe508del-CFTR from the PM^20^.

Here, we investigated whether HGF treatment would enhance the functional correction of Phe508del-CFTR by the VX-809/VX-770 drug combination. For this, we used *in vitro* epithelium-like cellular models to determine what would be the cellular and functional consequences of a prolonged, phase II trial duration-consistent, combined treatment with VX-809/VX-770 and HGF. We found that prolonged co-administration of HGF significantly increased the Phe508del-CFTR functional rescue by VX-809/VX-770 in bronchial polarized monolayers, also preventing VX-770-mediated destabilization of PM-rescued channels. Intriguingly, we show that prolonged co-treatment with HGF strengthens the integrity of bronchial and intestinal epithelium-like cellular cultures, suppressing yet unreported cellular dedifferentiation effects of continued exposure to VX-809, which may relate to the drug’s adverse effects.

Our data strongly suggests that HGF co-administration might be beneficial for CF patients, particularly in the initial weeks of treatment, and in patients with severe lung disease, who suffered from more frequent and pronounced adverse drug effects.

## Results

### HGF enhances functional rescue of Phe508del-CFTR by acute VX-809/VX-770 co-treatment

**Figure 1:**
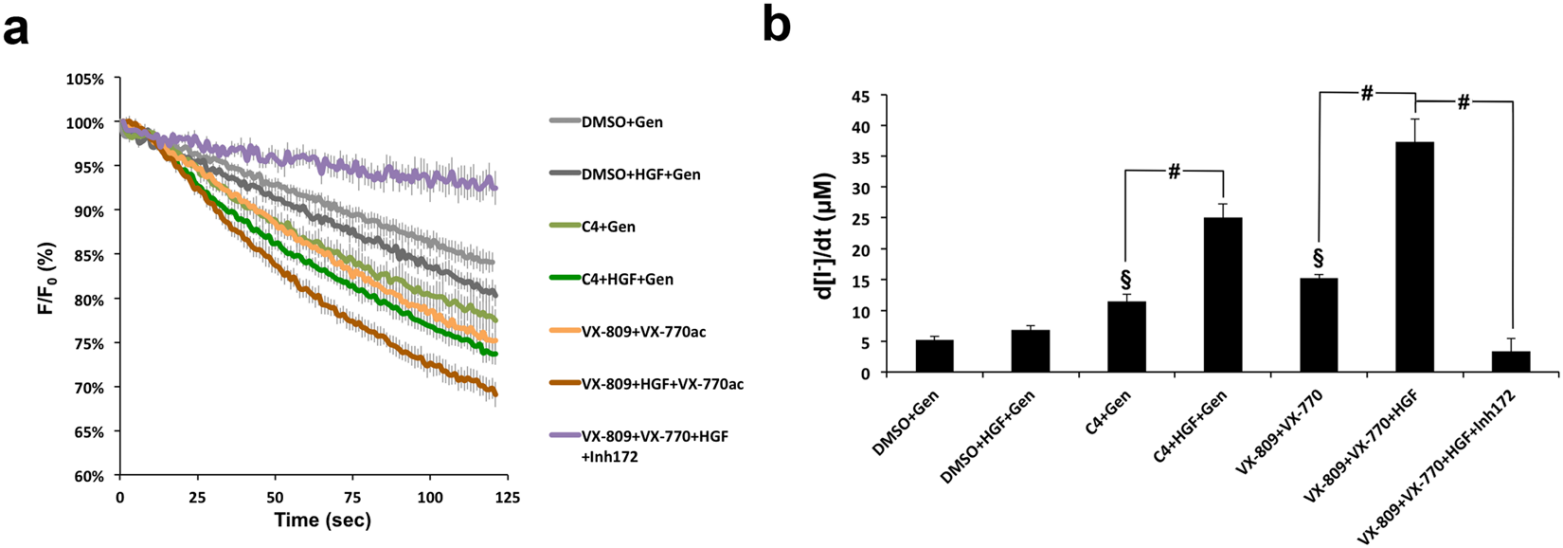
HGF treatment improves functional rescue of Phe508del-CFTR by chemical correctors. (**a**) Fluorescence decay curves of the iodide influx assay. CFBE-Phe508del cells stably expressing the YFP-halide sensor were treated, as indicated, for 48 h with 10 μM corr-4a (C4) or 3 μM VX-809, in the presence or absence of 50 ng/ml of HGF for the last 24 h. Cells were then stimulated with 5 μM forskolin (Fsk) and either 20 μM genistein (Gen) or 10 μM VX-770, in the presence or absence of 25 μM CFTR inhibitor 172 (inh172). Fluorescence was recorded continuously in a microplate reader, first for 10 s (baseline) and then for 110 s after the rapid (≤1 s) addition of isomolar PBS, in which Cl^−^ was replaced by I^−^. Fluorescence (F) was plotted over time as percentage of fluorescence at time 0 (F_0_). Data are means ± SEM of three independent assays. (**b**) Iodide influx rates calculated by fitting the curves to the exponential decay function to derive the maximal slope that corresponds to initial influx of I^−^ into the cells^23^. Data are means ± SEM of three independent assays. ^§^*p* < 0.01 relative to DMSO; ^#^*p* < 0.001.

We previously demonstrated that the functional correction of Phe508del-CFTR by corrector C4 in CFBE cells can be enhanced up to 3-fold by co-treatment with HGF^20^. Thus, we asked if HGF treatment would also enhance Phe508del-CFTR functional rescue when co-administered with the VX-809/VX-770 drug combination. To address this question we used the well-established halide-sensitive YFP (HS-YFP) functional assay^21,23,24^. Briefly, CFBE cells co-expressing Phe508del-CFTR and the halide-sensitive YFP-F46L/H148Q/I152L mutant^23^ (HS-YFP) were incubated in duplicates for 48 h with either DMSO, 10 μM of C4, or 3 μM VX-809. One of each replicate was co-treated with 50 ng/mL of HGF for the last 24 h. One additional replicate was added for the VX-809/HGF treatment, which was incubated with 25 μM of CFTR inhibitor 172 (inh172), 15 min prior to the assay. Cells were then stimulated for 30 min in PBS with 5 μM forskolin (Fsk), and either 20 μM genistein (Gen) or 10 μM VX-770. Analysis of HS-YFP fluorescence decay showed that HGF enhanced the functional response of C4-rescued Phe508del-CFTR by ~2.2-fold in this assay (Fig. 1a,b), in agreement with our previous findings using other methods^20^. Importantly, we also observed an equivalent increase (~2.5-fold) in CFTR function with VX-809/HGF co-treatment, versus VX-809 treatment alone, upon acute stimulation with Fsk and VX-770. Moreover, confirming a CFTR-specific response, co-treatment with inh172 reversed this effect (Fig. 1b).

**Figure 2:**
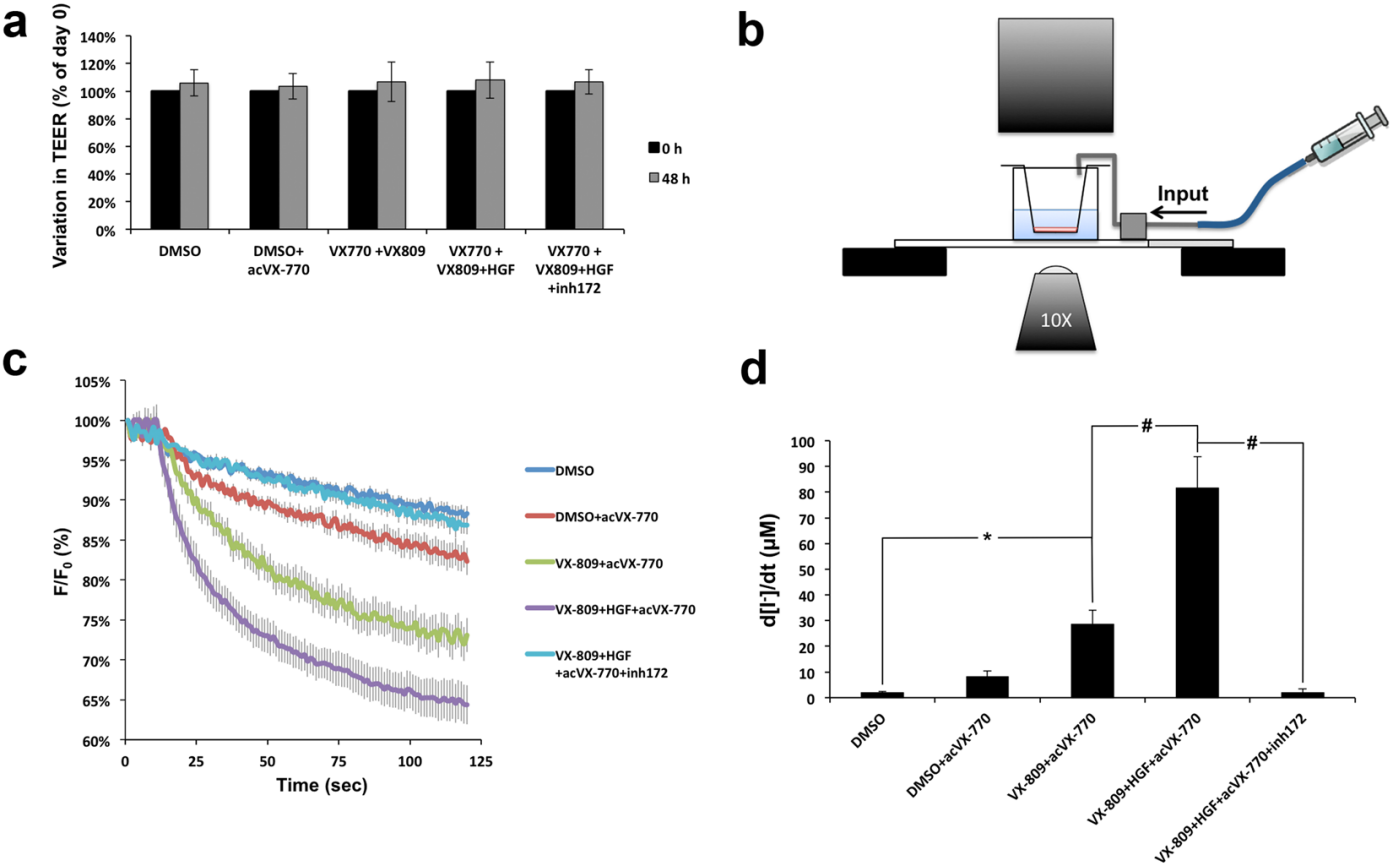
HGF treatment improves functional rescue of Phe508del-CFTR by VX-809/VX-770 in polarized CFBE cells. (**a**) Variation in TEER of polarized CFBE-Phe508del/HS-YFP cells treated for 48 h as indicated. (**b**) Schematic representation of the in-house setup used to record HS-YFP fluorescence decay in polarized cells. (**c**) Fluorescence decay curves of the iodide influx assay in polarized CFBE-Phe508del/HS-YFP cells, treated for 48 h with either DMSO or 3 μM of VX-809 in the presence or absence of HGF for the last 24 h, as indicated, and stimulated acutely with 10 μM of VX-770 (acVX-770). Fluorescence (F) was plotted over time as percentage of fluorescence at time 0 (F_0_). Data are means ± SEM of three independent assays. (**d**) Iodide influx rates calculated as in Fig. 1b. Data are means ± SEM of three independent assays. ^*^p < 0.01; ^#^*p* < 0.001.

Next, we tested whether the effect of HGF co-treatment was sustained in polarized CFBE cells. CFBE-Phe508del/HS-YFP cells were polarized in transwell, 0.4 μm PET filter inserts (24-well size), until they reached a transepithelial electrical resistance (TEER) of ≥600 Ω. Cells, in duplicate or triplicate filters, were treated with DMSO or 3 μM VX-809 for 48 h. Two of the VX-809 replicates were co-treated with 50 ng/mL of HGF for the last 24 h. One of these replicates was additionally incubated for 15 min with 25 μM of inh172 prior to the iodide influx assay. TEER was monitored for all conditions and no significant variations were observed over time (Fig. 2a).

**Figure 3:**
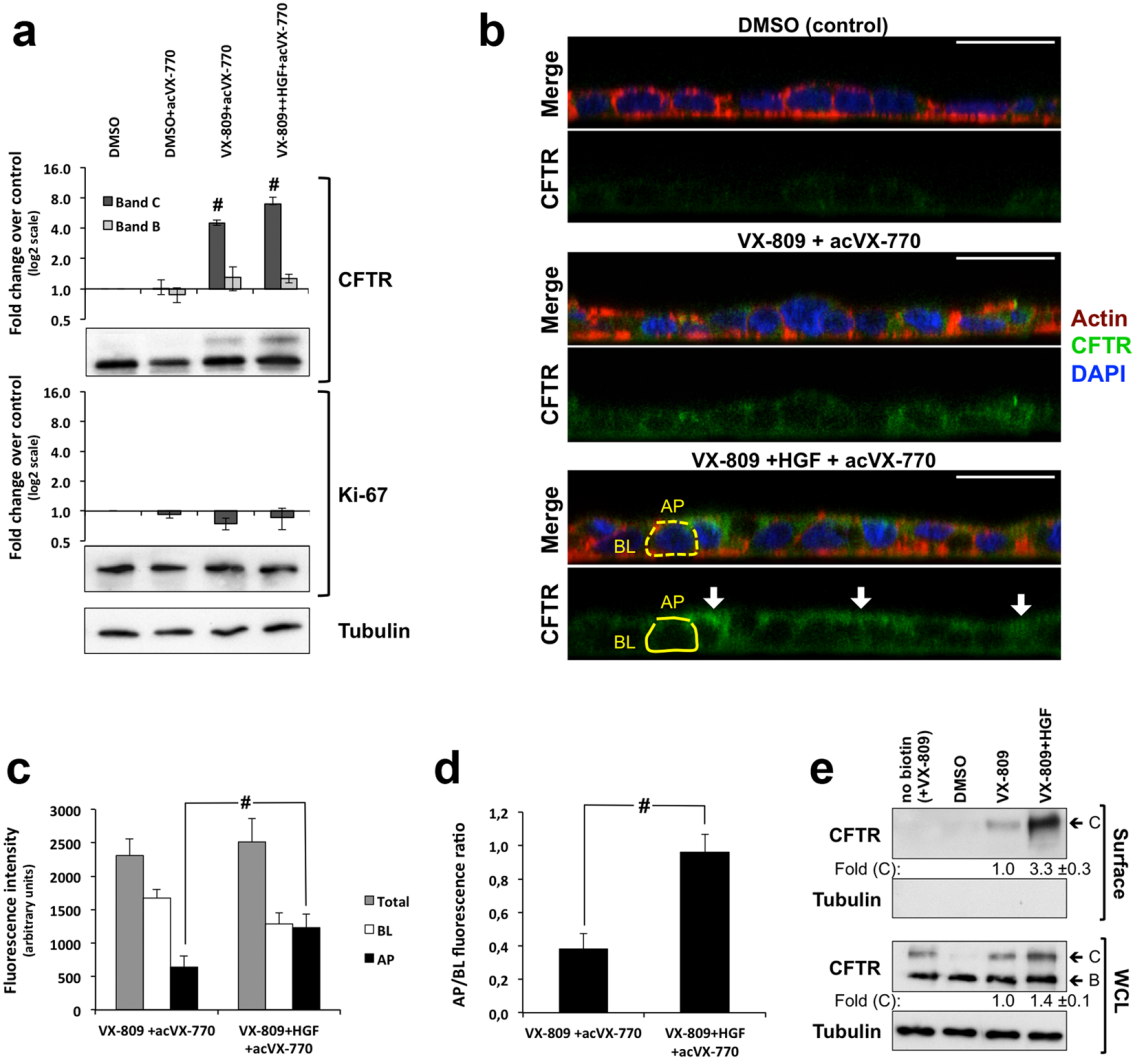
HGF treatment promotes apical localization of VX-809-rescued Phe508del-CFTR in polarized CFBE cells. (**a**) WB analysis of whole cell lysates from polarized CFBE cells treated for 48 h with either DMSO or 3 μM of VX-809 in the presence or absence of HGF for the last 24 h, as indicated, and stimulated acutely with 10 μM of VX-770 (acVX-770). Shown are representative images of CFTR and Ki-67 immunoblots together with bar plots of band intensity quantification, normalized to DMSO (means ± SEM), from three independent assays. Tubulin was used as a loading normalizer in band intensity quantification. (**b**) Immunofluorescence staining of polarized CFBE-Phe508del cells treated as in (a), were stained with anti-CFTR/Alexa 488, phalloidin-TRITC and DAPI, and analysed by confocal microscopy. Shown are overlay images (upper images) as well as isolated CFTR-staining (green channel) representative of the indicated treatment conditions. Yellow trace lines exemplify the method used for CFTR signal quantification. Actin signal was used as a guide to define the apical (AP) and basolateral (BL) membrane regions (dotted lines) that were used to quantify CFTR signal intensity (solid lines). AP, BL and Total (BL + AP) signal intensities in cells treated or not with HGF and VX-809/acVX-770 are plotted in (**c**) and the ratio between AP and BL signal intensities in (**d**). Data indicates mean ± SEM of signals from at least 30 cells analysed in each of the three independent experiments. White arrows indicate apical shift in CFTR localization upon HGF treatment. White horizontal bar represents 10 μm. ^#^*p* < 0.001 (**e**) Conventional cell surface protein biotinylation assays of CFBE cells treated as in (**a**). Fold change in total (WCL) and PM (Surface)-rescued Phe508del-CFTR band C levels were quantified by WB densitometry and are indicated below the respective panels.

To measure fluorescence decay after CFTR stimulation we developed an in-house setup (see *Methods* and Fig. 2b) that forms a chamber on top of a glass slide. The setup can be positioned on the confocal microscope stage and loaded with transwell inserts containing CFBE-Phe508del/HS-YFP polarized cells. This places the polarized cells within the focal length of a 10× objective (Fig. 2b), allowing live imaging acquisition. Thus, XZ confocal fluorescence images were collected continuously after the rapid (≤1 s) apical addition of iodide (I^−^) together with 5 μM Fsk, with or without 10 μM VX-770, and with or without 25 μM of inh172, as indicated (Fig. 2c, and see also Supplementary Movies SM1–SM5 for representative YFP quenching recordings). Calculation of initial iodide influx rates from the fluorescence decay curves (Fig. 2d) clearly showed that HGF co-treatment significantly increased the Phe508del-CFTR functional rescue by the combination of VX-809-mediated correction and VX-770 acute potentiation. Moreover, consistent with the results from non-polarized cells, this functional increase was 2.9-fold higher than with VX-809/VX-770 treatment alone and was completely suppressed by the presence of inh172 (Fig. 2d). Western blot (WB) analysis from these filters showed that HGF treatment produced a small increase in rescued Phe508del-CFTR mature C band steady-state levels (Fig. 3a), but this was not sufficient to justify the roughly 3-fold increase in CFTR-dependent ion transport (Fig. 2d). We, therefore, immunostained replicate filters to detect the localization of CFTR (Fig. 3b). We found that, more than enhancing the overall levels of CFTR in these cells, HGF promoted the accumulation of 2-times more rescued Phe508del-CFTR at the apical membrane (Fig. 3c), shifting to ~2.6-fold the ratio of CFTR apical/basolateral distribution (Fig. 3d). These data were confirmed by conventional cell surface protein biotinylation assays, where HGF produced a 1.4-fold increase in the total levels of VX-809-rescued band C but a 3.3 fold increment in the levels of CFTR at the PM (Fig. 3e). Both results are consistent with the enhancement of the channel’s anchoring and retention at the cell surface that we previously described for HGF co-treatment in C4-corrected CFBE-Phe508del cells^20^.

### Prolonged co-treatment with HGF sustains enhanced Phe508del-CFTR functional rescue by VX-809/VX-770 drug combination

The hyperactivity of the HGF/c-MET pathway has been observed in numerous neoplasms, including non-small-cell lung carcinomas^25^. Prolonged or continuous activity of the c-MET receptor by mutation, gene amplification, or altered signalling properties, leads to excessive cell proliferation and is related to neoplastic disease development and progression^26^.

**Figure 4:**
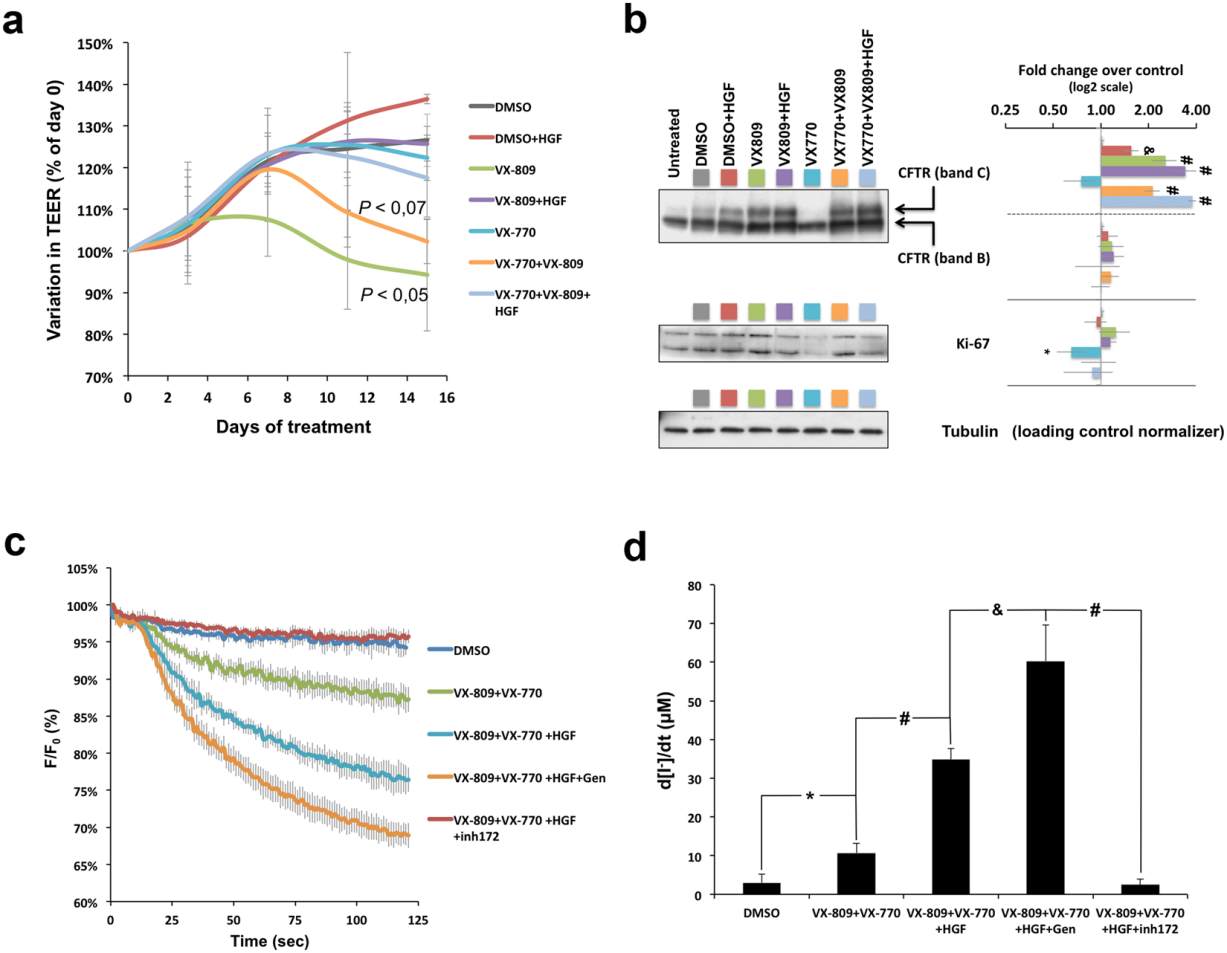
Prolonged HGF treatment improves functional rescue of Phe508del-CFTR by the VX-809/VX-770 combination without stimulating proliferation of polarized CFBE cells. (**a**) Variation in TEER of polarized CFBE-Phe508del cells treated for 15 days with DMSO, HGF (50 ng/ml), VX-809 (3 μM), VX-770 (0.5 μM) alone or in the indicated combinations. Conditions leading to significant or near-significant TEER variations are indicated. (**b**) WB analysis of whole cell lysates from polarized CFBE-Phe508del cells treated as in (**a**). Shown are representative images of CFTR and Ki-67 immunoblots together with bar plots of band intensity quantification, normalized to DMSO (means ± SEM) from three independent assays. Tubulin was used as a loading normalizer in band intensity quantification. (**c**) Fluorescence decay curves of iodide influx assays in polarized CFBE-Phe508del/HS-YFP cells treated as in (**a**) and stimulated with 5 μM Fsk alone or together with 20 μM of Gen in the presence or absence of 25 μM of inh172, as indicated. Fluorescence (F) was plotted over time as percentage of fluorescence at time 0 (F_0_). (**d**) Iodide influx rates calculated as in Fig. 1b. Data are means ± SEM of three independent assays. ^*^p < 0.05, ^&^*p* < 0.005, and ^#^*p* < 0.001.

Thus, we used WB to analyse the levels of Ki-67 protein in the lysates of polarized CFBE-Phe508del cells, treated as above. Ki-67 is commonly and reliably used as a pro-proliferative marker for human cells and tumours^27^. It is present during all active phases of the cell cycle, being markedly increased in proliferating cell populations but only residual or absent in resting (non-dividing) cells^28^. Here, we observed no significant change in Ki-67 abundance in polarized CFBE-Phe508del cells after 24 h of HGF treatment (Fig. 3a). Therefore, we decided to analyse the effects of a more prolonged treatment with HGF, both regarding its enhancement of VX-809/VX-770 action and its effect on the differentiation and proliferation of polarized CFBE epithelia. We chose to extend our treatment for a period of 15 days, since this would be within the time frame of a phase II trial^8^. Moreover, we also considered previous studies reporting that the prolonged exposure to VX-770 concentrations above 1 μM inhibited the functional rescue of Phe508del-CFTR by VX-809^13,15^. Thus, CFBE-Phe508del cells were polarized in filter inserts to a minimum TEER value of 600 Ω and exposed, for 15 days, to either DMSO (control), DMSO + 50 ng/ml HGF, 3 μM VX-809, 3 μM VX-809 + 50 ng/ml HGF, 0.5 μM VX-770, 0.5 μM VX-770 + 3 μM VX-809, or 0.5 μM VX-770 + 3 μM VX-809 + 50 ng/ml HGF. TEER was monitored at days 3, 7, 11, and 15, and the culture medium replaced by fresh medium containing the same compound combinations. A clear decrease in relative TEER values (normalized to day 0) was observed from day 3 onwards in cells treated with VX-809 alone, and in cells treated with VX-809/VX-770 after day 7, when compared to DMSO (Fig. 4a). Notably, co-treatment with HGF prevented relative TEER decrease in CFBE cells treated either with VX-809 alone or in combination with VX-770 (Fig. 4a). WB analysis of these cells showed a significant ~1.5-fold increase in CFTR C band levels in cells treated with HGF alone (DMSO + HGF), compared to DMSO, consistent with an enhanced apical retention of the residual Phe508del-CFTR rescued by prolonged exposure to low DMSO concentrations (Fig. 4b).

Prolonged VX-809 treatment produced a significant ~2.5-fold increase in mature C band levels, compared to DMSO. Co-treatment with VX-809 and VX-770 also produced a significant increase in C band abundance, though slightly lower (~2.1-fold) than with VX-809 alone (Fig. 4b). Importantly, the effect of HGF was additive to the prolonged treatment with VX-809 alone (from ~2.5 to ~3.4-fold, over DMSO) and, remarkably, it appears to protect VX-809-rescued Phe508del-CFTR from the destabilization caused by VX-770 co-treatment, allowing for C band levels ~3.7-fold greater than DMSO-treated cells (Fig. 4b). No significant variations in B band levels were observed between treatments.

Next, we tested the double and triple 15-day treatments in CFBE cells co-expressing Phe508del-CFTR and the HS-YFP sensor. As before, fluorescence decay in these cells was measured after CFTR stimulation with 5 μM Fsk. Treatment with the VX-809/VX-770 combination produced a significant ~3.6-fold increase in iodide influx over DMSO treated cells (Fig. 4c,d), although in absolute values this activity was roughly 2.5 times lower than that achieved with 48 h of VX-809 treatment followed by acute 10 μM VX-770 potentiation (see Fig. 2d). Consistent with the observed impact on the apical retention of rescued CFTR, co-treatment with HGF more than tripled (~3.3-fold) the effect of the VX-drugs alone (Fig. 4d), a result that was reverted by the presence of 25 μM inh172. Notably, the effect of HGF co-treatment was increased by ~1.7-fold when 20 μM Gen and 5 μM Fsk were added acutely (Fig. 4c and d), consistent with further potentiation of apically rescued CFTR.

Remarkably, prolonged treatment with HGF, alone or in combination with the VX-drugs, did not increase the steady-state levels of Ki-67, which indicates no change in the proliferative behaviour of these cells (Fig. 4b). Of note, there was a small (1.5-fold) but significant decrease in the levels of Ki-67 upon prolonged exposure to VX-770 alone.

### Co-treatment with HGF prevents depolarization of CFBE epithelium-like monolayers after prolonged exposure to VX-809

**Figure 5:**
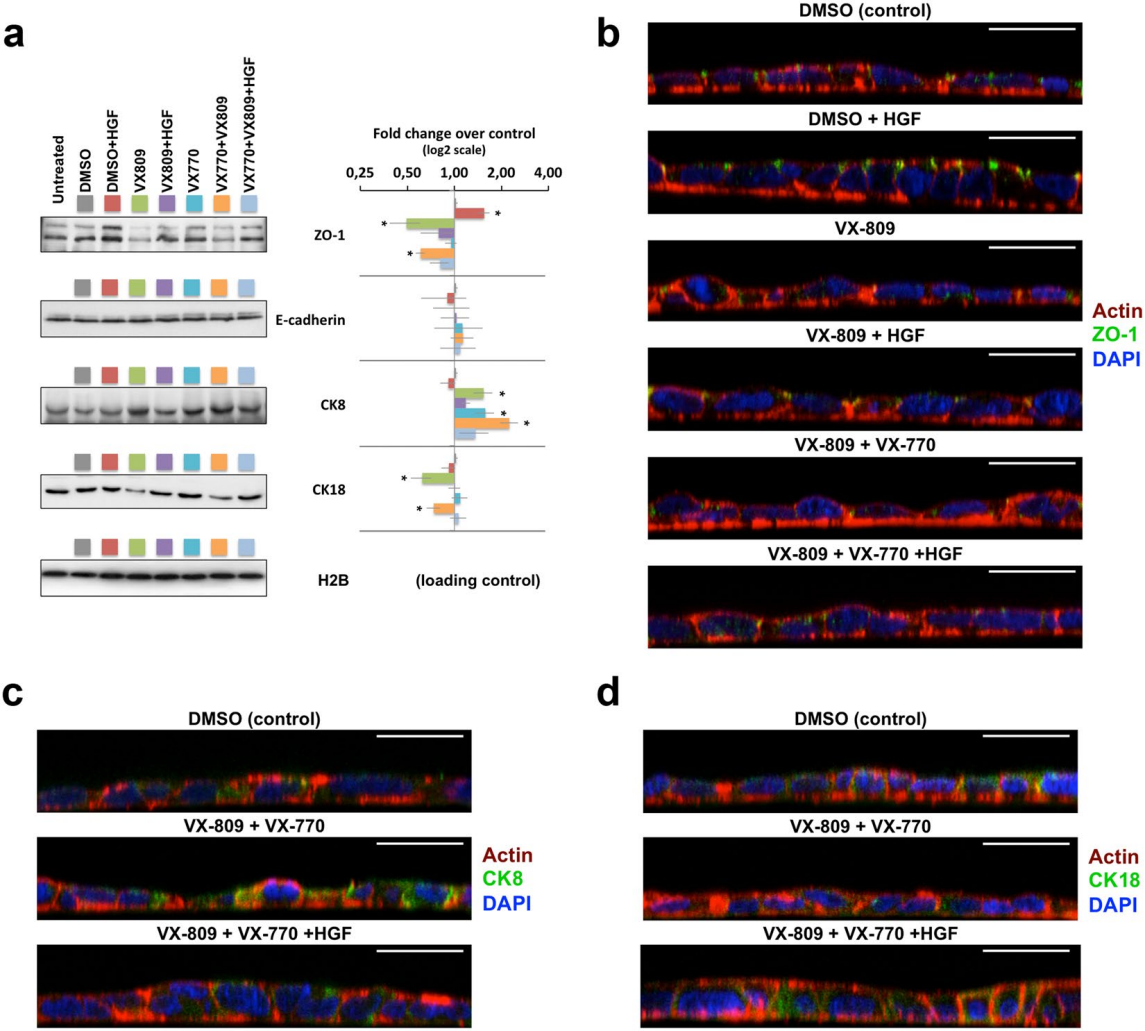
HGF co-treatment prevents dedifferentiation of polarized CFBE cells after prolonged exposure to VX-809. Polarized CFBE-Phe508del cells were treated for 15 days with DMSO, HGF (50 ng/ml), VX-809 (3 μM), or VX-770 (0.5 μM), alone or in the indicated combinations. (**a**) WB analysis of whole cell lysates. Shown are representative images of immunoblots for ZO-1, E-cadherin, CK8, and CK18 together with bar plots of band intensity quantification, normalized to DMSO (means ± SEM) from three independent assays. Histone H2B was used as a loading normalizer in band intensity quantification. (**b**–**d**) Immunofluorescence staining of polarized CFBE-Phe508del cells, treated as in (**a**), were stained with phalloidin-TRITC, DAPI, and either anti-ZO-1/Alexa 488 (**b**), anti-CK8/Alexa 488 (**c**), or anti-CK18/Alexa 488 (**d**), and analysed by confocal microscopy. Shown are overlay images representative of the indicated treatment conditions. White horizontal bars represent 10 μm. ^*^p < 0.05.

The decrease in TEER observed in polarized monolayers exposed to VX-809 for more than 3 to 7 days (see Fig. 4a) led us to use WB to analyse the effects of the prolonged treatment on epithelial integrity and differentiation markers (Fig. 5a). While E-cadherin levels showed no significant variations, a significant ~2-fold decrease in Zonula Occludens-1 (ZO-1) steady-state abundance was detected in both conditions where cells were treated with VX-809 (Fig. 5a).

Once again, co-treatment with HGF seemed to revert this effect in both cases, bringing ZO-1 levels back to those observed in DMSO control cells. In fact, incubation with HGF alone produced a small (~1.5-fold) but significant increase in ZO-1 levels, which might represent compensation for VX-809-induced ZO-1 downregulation. These effects were also observed through confocal microscopy after ZO-1 immunostaining (Fig. 5b). Moreover, we could confirm that treatment with HGF alone stimulated ZO-1 localization to sub-apical, focal cell-cell contacts, consistent with an enhancement of tight-junction (TJ) maturation. This observation, together with the clear morphologic improvement in cell polarity (as seen through phalloidin staining of the actin cytoskeleton - Fig. 5b) and the increase in relative TEER (Fig. 4a), is indicative that the prolonged HGF treatment of bronchial epithelial cells has a pro-differentiation effect. In contrast, treatment with VX-809 alone led to a clear loss of ZO-1 at TJ sites that, based on the actin cytoskeleton morphology, was accompanied by a loss of cellular polarity (Fig. 5b) consistent with the pronounced decrease in TEER observed after 15 days of treatment (Fig. 4a). Combination of VX-809 and VX-770 had a similar effect on ZO-1 levels and distribution at day 15, albeit with a lower impact on cellular morphology and TEER levels relative to VX-809 alone (Figs 4a and 5b, respectively), possibly related to the observed delay in TEER decline induced by VX-770 co-treatment (Fig. 4a). Importantly, co-incubation with HGF clearly reverted VX-809-induced CFBE depolarization, particularly in the triple treatment combination, where the cellular phenotype closely resembled that of DMSO-treated cells (Fig. 5b). We also analysed the steady-state levels of cytokeratin 8 (CK8) and 18 (CK18) after the 15-day treatment. Interestingly, we found that whereas the prolonged treatment with VX-809 significantly decreased the steady-state abundance of CK18, either alone or in combination with VX-770 (Fig. 5a), CK8 levels increased in response to both VX-809 and VX-770. Moreover, the combination of the two drugs had an additive effect, increasing CK8 levels ~2.3-fold over DMSO-treated cells (Fig. 5a). Importantly, co-treatment with HGF returned CK8/CK18 expression to near control levels, even in the presence of the VX-809/VX-770 combination (Fig. 5a,c,d).

### HGF treatment prevents depolarization of colorectal epithelial cells after prolonged exposure to VX-809/VX-770 treatment

Several cases of off-target effects have been reported for VX-809/VX-770 combination treatment, most of which result in pulmonary and gastrointestinal complications^29^. Given the observed effects in the polarization of bronchial epithelial cells, we questioned the consequences of a prolonged treatment with the VX-drugs, alone and in combination with HGF, on the polarity and expression of epithelial differentiation markers in cells from gastrointestinal origin.

**Figure 6:**
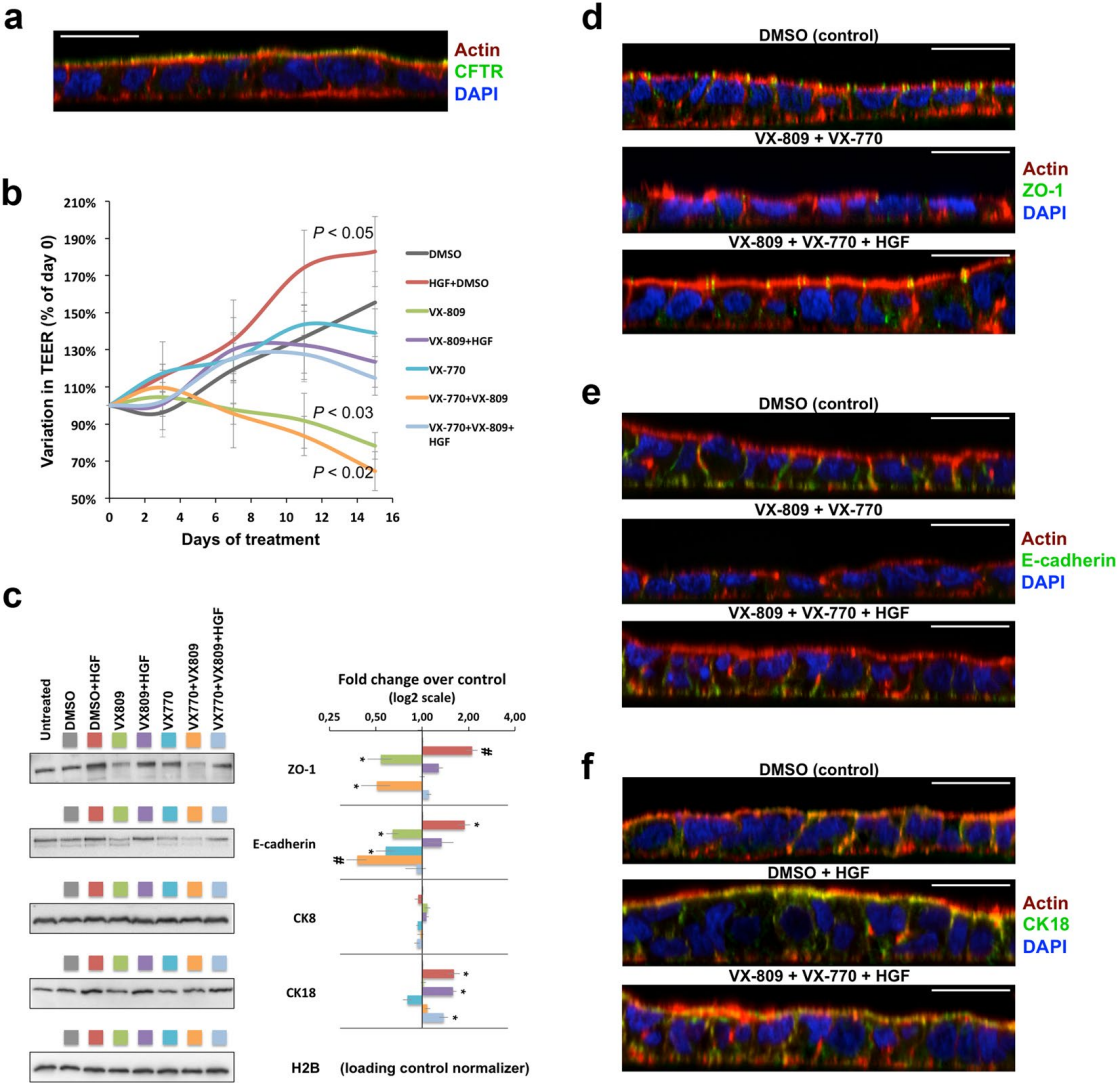
HGF co-treatment prevents dedifferentiation of polarized Caco-2 cells after prolonged exposure to the VX-809/VX-770 drug combination. (**a**) Caco-2 colorectal cells were polarized to a TEER above 600 Ω, stained with phalloidin-TRITC, DAPI, and anti-CFTR/Alexa 488, and analysed by confocal microscopy. (**b**) Variation in TEER of polarized Caco-2 colorectal cells treated for 15 days with DMSO, HGF (50 ng/ml), VX-809 (3 μM), or VX-770 (0.5 μM), alone or in the indicated combinations. Conditions leading to significant TEER variations are indicated. (**c**) WB analysis of whole cell lysates from polarized Caco-2 cells treated as in (**b**). Shown are representative images of immunoblots for ZO-1, E-cadherin, CK8, and CK18 together with bar plots of band intensity quantification, normalized to DMSO (means ± SEM) from three independent assays. Histone H2B was used as a loading normalizer in band intensity quantification. (**d**–**f**) Immunofluorescence staining of polarized Caco-2 cells, treated as in (**b**), with phalloidin-TRITC, DAPI, and either anti-ZO-1/Alexa 488 (**d**), anti-E-cadherin/Alexa 488 (**e**), or anti-CK18/Alexa 488 (**f**) and analysed by confocal microscopy. Shown are overlay images representative of the indicated treatment conditions. White horizontal bars represent 10 μm. ^*^p < 0.05; ^#^*p* < 0.01.

The human intestinal Caco-2 cell line has been extensively used over the last decades as a model for the intestinal epithelium, namely to study the regulation and function of wild-type CFTR, which these cells express endogenously and at high levels (see Fig. 6a)^30–34^. Originally obtained from a human colon adenocarcinoma, these cells undergo a process of spontaneous differentiation in culture that leads to the formation of a cell monolayer, resembling a simple columnar epithelium (Fig. 6a), which exhibits several morphological and functional characteristics of mature enterocytes^34,35^. Thus, as before, we polarized Caco-2 cells in filter inserts to a TEER of ~600 Ω and exposed them for 15 days to either DMSO (control), DMSO + 50 ng/ml HGF, 3 μM VX-809, 3 μM VX-809 + 50 ng/ml HGF, 0.5 μM VX-770, 0.5 μM VX-770 + 3 μM VX-809, or 0.5 μM VX-770 + 3 μM VX-809 + 50 ng/ml HGF. As for CFBE cells, TEER was monitored at days 3, 7, 11, and 15, and the cell medium was replaced by fresh medium containing the same compound combinations.

A significant decrease in relative TEER (normalized to day 0) was observed from day 3 onwards in cells treated with VX-809, either alone or in combination with VX-770, when compared to DMSO-treated cells (Fig. 6b). Notably, in both situations, co-treatment with HGF obviated the decrease in relative TEER (Fig. 6b). Moreover, treatment with HGF alone seemed to promote Caco-2 differentiation, significantly increasing TEER values when compared to control cells. WB analysis showed that HGF alone promoted a ~2-fold increase in ZO-1 and E-cadherin levels, two markers of epithelial differentiation (Fig. 6c). In contrast, prolonged exposure to VX-809, alone or in combination with VX-770, led to a nearly 2-fold decrease in the steady-state abundance of ZO-1, similar to the observed with CFBE cells. In both cases, co-incubation with HGF reverted ZO-1 decrease by the VX-drugs (Fig. 6c). Consistently, immunostaining of Caco-2 cells showed that prolonged exposure to VX-drugs results in a clear dedifferentiation of epithelial-like morphology, with abrogation of ZO-1 expression and localization, leading to an apparent loss of TJs integrity (Fig. 6d). Moreover, in contrast to what was observed in CFBE cells, prolonged treatment of Caco-2 cells with either VX-809 or VX-770 alone, resulted in a clear (>1.6-fold) decrease of E-cadherin steady-state levels, producing a nearly 3-fold decrease when both drugs were combined (Fig. 6c). This effect on E-cadherin was also readily seen by confocal microscopy after immunostaining of Caco-2 cells (Fig. 6e). Notably, co-treatment with HGF prevented E-cadherin downregulation by the VX-drug combination (Fig. 6c), restoring TEER values (Fig. 6b), E-cadherin localization to cell-cell contacts, and polarized epithelium morphology (Fig. 6e). Interestingly, Caco-2 cells did not show any significant variation in CK8 expression in response to the different treatments (Fig. 6c). In addition, while a small decrease in CK18 levels was observed upon prolonged exposure to VX-770 (not statistically significant), HGF treatment produced a significant ~1.6-fold increase in CK18 expression, which was sustained in the presence of both VX-drugs (Fig. 6c). This effect was also clearly observed in immunostained cells (Fig. 6f).

### HGF and the VX-809/VX-770 combination have opposite effects on Ki-67 expression in Caco-2 cells

**Figure 7:**
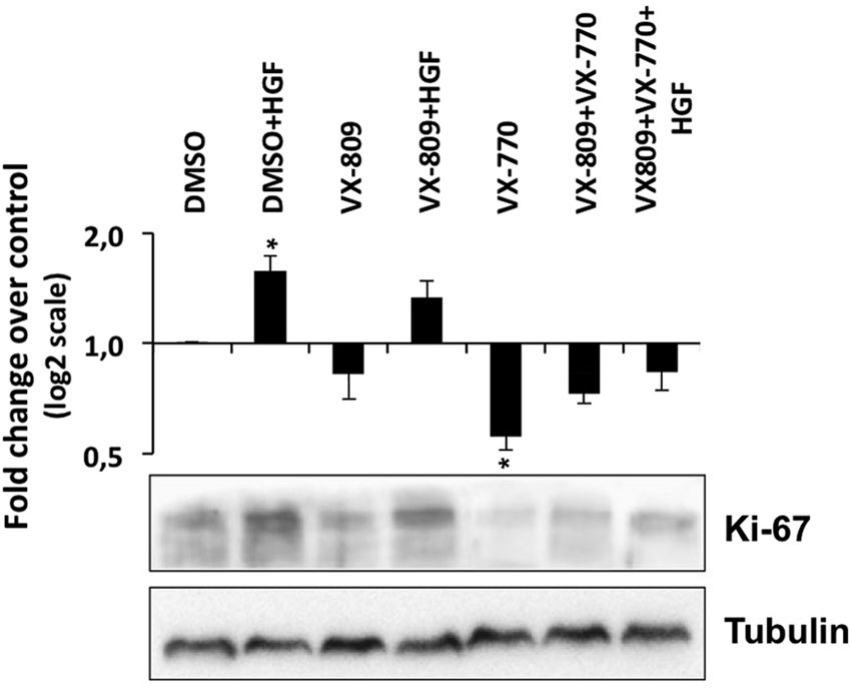
Prolonged HGF and VX-770 treatments have opposite effects on Ki-67 levels in polarized Caco-2 cells. Polarized Caco-2 colorectal cells were treated for 15 days with DMSO, HGF (50 ng/ml), VX-809 (3 μM), or VX-770 (0.5 μM), alone or in the indicated combinations. Shown are representative WB images of whole cell lysates probed with anti-Ki-67 and anti-Tubulin antibodies. Bar plot shows quantification of Ki-67 band intensity, normalized to DMSO (means ± SEM) from three independent assays. Tubulin was used as a loading normalizer in band intensity quantification.

Analysis of Ki-67 steady-state abundance in polarized Caco-2 cells revealed that whereas stand-alone exposure to HGF significantly increased the levels of this pro-proliferative marker (~1.6-fold), incubation with the VX-770 had the opposite effect, resulting in a ~1.8-fold decrease in Ki-67 expression (Fig. 7). Significantly, the triple combination (VX-809/VX-770/HGF) resulted in Ki-67 levels close to those of DMSO-treated cells, consistent with a reversal of VX-770- induced Ki-67 downregulation by HGF co-treatment (Fig. 7).

## Discussion

In this study we investigated three major questions: (1) whether HGF treatment would enhance the functional correction of Phe508del-CFTR by the VX-809/VX-770 drug combination; (2) whether the effect would be sustained in polarized, epithelium-like bronchial cell monolayers; and (3) what would be the cellular and functional consequences of a phase II trial duration-consistent, 15-day long combined treatment with VX-809/VX-770 and HGF.

We found, similar to what we had previously shown for corrector C4^20^, that 24 h of co-treatment with HGF increased by roughly 3-fold the functional correction of Phe508del-CFTR that can be achieved in CFBE cells by a 48 h treatment with VX-809 followed by acute potentiation with VX-770. Significantly, this result was consistently observed in both polarized and non-polarized cells. Moreover, we found evidence that HGF treatment also significantly enhanced the apical accumulation of VX-809-rescued Phe508del-CFTR in polarized CFBE cells, not by varying the total abundance of the protein but by increasing the ratio of its apical/basolateral distribution, indicating an enhanced retention of the rescued channels at the cell’s apical membrane. This is consistent with our previous findings that HGF treatment stimulates the anchoring of apical CFTR to the actin cytoskeleton via stimulation of RAC1-mediated PIP2 production and the formation of a CFTR/NHERF1/Ezrin/F-actin macromolecular complex^20^. Importantly, we found that the effect of HGF treatment was still observable after prolonged, 15-day co-treatment with HGF, VX-809 and 0.5 μM VX-770. Notably, prolonged exposure to this low VX-770 concentration still decreased VX-809-mediated rescue of Phe508del-CFTR. This is consistent with previous findings that sustained exposure to VX-770, even at concentrations below 1 μM, can interfere with Phe508del-CFTR cell surface stability, promoting its PM removal, accelerating its internalization and markedly increasing its turnover rate^13,14^. Also of note, we show here that HGF can protect VX-809-rescued Phe508del-CFTR from VX-770 induced destabilization. This is in agreement with our previous findings that HGF treatment prevents the internalization of rescued Phe508del-CFTR by promoting its anchoring to the sub-apical cytoskeleton^20,21^, and with our current observation that HGF exposure leads to the apical accumulation of VX-809/VX-770-rescued CFTR. Moreover, whereas the functional output of VX-809-rescued Phe508del-CFTR after prolonged treatment with 0.5 μM VX-770 was considerably lower than that achieved by acute potentiation with 10 μM of the drug, co-treatment with HGF more than compensated for this decrease, reaching levels of CFTR-mediated ion transport 30% higher than those of the acute treatment. In addition, this effect was nearly doubled by acute co-stimulation with genistein, which is consistent with previous reports that VX-809-rescued CFTR channels at the apical membrane are poorly potentiated by therapeutic range VX-770 concentrations^15^, making them receptive to further potentiation^13,15^. Consistently, a recent study showed that potentiators, such as genistein and curcumin, additively enhanced forskolin-induced swelling of VX-809/VX-770-treated intestinal organoids derived from biopsies of Phe508del homozygous patients^36^. Together with our results, these data suggest that genistein and/or curcumin could be used in combination with HGF to synergistically increase CFTR-mediated chloride secretion in the treatment of CF.

The VX-809/VX-770 combination has now been used in patients since 2015, and several cases of off-target side-effects have been reported^29^. These adverse effects can manifest in up to 40% of treated patients, with the vast majority (~80%) being pulmonary but with some patients (~24%) also manifesting gastrointestinal symptoms, including diarrhoea and nausea^12^. Our findings suggest that these manifestations may, at least partially, be related to a previously unreported epithelial dedifferentiation effect resulting from prolonged exposure to VX-809. This is of particular interest since we show that HGF treatment was highly effective in preventing VX-809-induced dedifferentiation of bronchial and colorectal epithelium-like monolayers. In polarized bronchial epithelia cells, we demonstrated that HGF restored ZO-1 levels, TJ localization and TEER values. It is interesting to note that VX-770 also had a partially protective effect on VX-809-induced dedifferentiation of CFBE cells, delaying TEER reduction by 3 days when compared to VX-809 stand-alone treatment. This protective effect of VX-770 may relate to its impact on Ki-67 levels. We observed that prolonged exposure to VX-770 alone nearly depleted Ki-67 levels in CFBE cells. Absence of Ki-67 is associated with resting cells that have entered a ‘quiescent’ state due to the absence of extrinsic pro-proliferative signals^37–39^. Our results indicate that prolonged exposure to low concentrations of VX-770 may somehow mimic this state, partially desensitizing bronchial epithelial cells from depolarization by VX-809.

HGF also prevented VX-809-induced dedifferentiation of colorectal Caco-2 polarized cells. For this cell type, however, although prolonged treatment with VX-770 also significantly decreased Ki-67 abundance, it was not sufficient to constrain the depolarizing effect of VX-809 when used in combination. In fact, the VX-drug combination produced the most pronounced TEER decrease in Caco-2 cells, which by the end of the treatment reached below 65% of the initial value (corresponding to TEER values under 400 Ω). This may relate to our observation that stand-alone treatment with either drug produced a significant decrease of E-cadherin steady-state levels, which was additive, reaching nearly 3-fold when both drugs were combined. Indeed, decreased expression of E-cadherin and ZO-1 adhesion molecules are well known markers of colorectal cell dedifferentiation and strongly correlate with epithelial-mesenchymal transition (EMT) in colorectal cancers^40–42^. Importantly, despite inducing a small but significant increase in Ki-67 levels when used alone, prolonged treatment with HGF had a marked pro-differentiation effect on polarized colorectal cell monolayers; it stimulated ZO-1 and E-cadherin expression, promoted their localization to cell-cell contacts, prevented their downregulation by VX-drugs, and restored TEER values and epithelial morphology. Moreover, the opposite effects of HGF and VX-drugs on Ki-67 expression apparently compensate for each other, producing a net result of Ki-67 levels close to those of control cells.

We also monitored the levels of CK8 and CK18. These intermediate filament components are typically co-expressed as the primary keratin pair in simple epithelial cells, but tissue-specific variations in their expression are often associated with cancer-related cellular dedifferentiation and EMT^43,44^. In lung cancers, for instance, increased CK8 expression is correlated with increased invasiveness of tumour cells *in vitro* and *in vivo*^45^. In contrast, the role of CK18 upregulation is less clear, having been described associated with lung cancer progression^46^, as well as with better differentiation and decreased tumour malignancy^47^. Here we found that the dedifferentiation of CFBE cells induced by prolonged exposure to VX-809 associated with a significant increase in CK8 levels and a decrease in CK18 expression. Interestingly, whereas prolonged exposure to VX-770 alone also increased CK8 abundance, being additive with VX-809 in the combination treatment, it did not, however, decrease the levels of CK18. Rather, co-treatment with VX-770 reduced the downregulation of CK18 by VX-809, which considering that the co-treatment delayed TEER decrease in CFBE cells, is consistent with the reported effect of CK18 in preventing dedifferentiation of lung epithelial cells^47^. Again, co-treatment with HGF attenuated the variations in CK8/18 levels induced by the VX-drugs, consistent with its effect in restoring epithelial morphology. Notably, in colorectal Caco-2 cells, neither VX-809 nor VX-770, alone or in combination, induced significant variations in CK8/18 levels. Loss of CK8 expression has been associated with hyperproliferation of colorectal cells^48,49^, but despite elevating Ki-67 levels, prolonged HGF treatment produced no significant change in CK8 levels in polarized Caco-2 cells. However, HGF significantly upregulated CK18 levels. Upregulation of CK18 levels has been associated with promotion of enterocytic differentiation of Caco-2 cells, via Notch signalling^49^, and this is consistent with previous reports showing that exposure to HGF accelerates Caco-2 cell differentiation by stimulating the metabolic and structural events accompanying this process^50^.

In summary, we found that, as expected, HGF treatment also significantly enhanced the functional rescue of Phe508del-CFTR by acute treatment with the VX-809/VX-770 drug combination. Moreover, we further demonstrated that this enhancement was sustained in polarized bronchial epithelial monolayers after prolonged, 15-day HGF co-treatment with VX-809 and low, therapeutic-compatible VX-770 doses, reaching functional rescue levels higher than those achieved by acute potentiation with high VX-770 concentrations. Importantly, we found that HGF treatment also prevented VX-770-mediated destabilization of rescued Phe508del-CFTR and enabled further potentiation of the rescued channels by genistein, indicating that HGF co-treatment would also favour previously suggested combination therapies using multiple correctors^36^. Most striking, we observed that rather than promoting cell scattering and proliferation, a well-known effect of HGF on polarized MDCK cells^51^, prolonged HGF treatment of polarized airway cells actually prevented the previously unrecognized epithelial dedifferentiation effects of prolonged exposure to VX-809. While the physiologic relevance of these observations cannot be fully ascertained through the use of *in vitro* models, they strongly suggest that there may be a potential gain in addressing *in vivo* and *ex vivo* (in patient-derived tissues), the effects of adding HGF to current combinational drug therapies. Moreover, while polarized CFBE and Caco2 monolayers do not fully recapitulate the properties of differentiated epithelia *in vivo*, these models have been extensively used to study the mechanisms of CF disease and, in the case of CFBE cells, for the development and characterization of several CFTR modulator drugs^13–15,52,53^, where they have been shown to respond similarly to primary airway cells^20,52,53^. Thus, our finding of previously unreported potentially dedifferentiative effects of prolonged exposure to VX-809 in these cells highlights the importance of using more extended treatment periods, while assessing the cellular effects of new CF-modulator drugs in pre-clinical studies. It should be mentioned that a next-generation CFTR corrector, VX-661 (Tezacaftor), in combination with VX-770, has proven to be equally effective and better tolerated than VX-809 in clinical trials^54–56^, and recently received FDA approval for the treatment of patients, aged 12 years and older, homozygous for the Phe508del mutation or having at least one CFTR mutation that is responsive to the drug combination, based on *in vitro* data and/or clinical evidence^57^. However, it should be noted that the destabilizing effect of long exposure to VX-770 therapeutic-range concentrations on VX-661-rescued Phe508del-CFTR channels was even more pronounced than that observed for VX-809^14^. This suggests that, if supported by further *in vivo* studies, the potentially protective effect of HGF co-treatment against VX-770-induced destabilization of apically rescued Phe508del-CFTR could also be beneficial for VX-661-treated patients. This might be even more applicable in the case of patients with severe lung disease (predicted FEV_1_ < 40%) that were not included in the initial VX-661/VX-770 trials^54–56^, but for which recent clinical data, following treatment with VX-809/VX-770, revealed a considerably higher frequency and severity of therapy-related respiratory adverse events (particularly in the first weeks of treatment) and lessened clinical benefit^10,58,59^. Supportive of a potential application of HGF in the CF setting, several studies in animal models have provided strong evidence that HGF administration potently mitigates the effects of acute and chronic lung injuries caused by oxidative stress and inflammation^60^. Encouragingly, some of these studies have also shown that HGF had a protective effect when given either simultaneously with or after prolonged lung-damaging stimuli, suggesting that HGF pro-regenerative action is effective during both the initiation and the progressive phase of lung disease^61^.

Taken together, our findings emphasise the critical importance of assessing the cellular effects of prolonged exposure to investigational CF drugs in pre-clinical studies and provide compelling evidence for the potential benefit of including HGF in CF combination therapies, alerting the scientific community to the need and relevance of further *in vivo* studies with this physiological factor in the context of CF. Supporting the plausibility of such studies, the intravenous/systemic administration of recombinant HGF protein has been well tolerated in phase I/II clinical trials for liver regeneration^62^ and it is currently under trial for its regenerative effects in several other conditions^63,64^.

## Methods

### Cell culture, polarization and treatment

CFBE41o- cells stably expressing Phe508del-CFTR (CFBE-Phe508del cells) (a kind gift from JP Clancy, University of Alabama USA) were maintained in minimal essential medium (MEM) supplemented with L-glutamine, Earle’s salts, 10% (v/v) foetal bovine serum (FBS) and 2 μg/ml puromycin, whereas Caco-2 cells were maintained in RPMI supplemented with 10% (v/v) FBS (all reagents were from Life Technologies Invitrogen Corporation). All cell lines were cultured at 37 °C under 5% CO_2_ and regularly checked for the absence of mycoplasm infection. CFBE-Phe508del cells stably co-expressing the YFP-F46L/H148Q/I152L halide sensor (CFBE-Phe508del/HS-YFP CFBE cells) were generated by transfection with LipofectAMINE 2000 followed by 3-week selection with 0.4 mg/ml hygromycin B (both from Thermo Scientific). Fluorescent cells were then sorted (FACSAriaIII, BD Biosciences) and expanded maintaining selective pressure with 0.4 mg/ml hygromycin B.

Cell monolayers were polarized in transwell, collagen IV coated, porous (0.4 μm) PET filter inserts (Ø 6.4 mm, from Falcon – Thermo Fisher Scientific), in medium supplemented with 2.5% FBS, until they reached a transepithelial electrical resistance (TEER) above 600 Ω, as measured with a Chopstick Electrode (STX2 from WPI®). Cells were then treated for the indicated periods, with DMSO (Sigma Aldrich) or the described concentrations of recombinant human HGF (Santa Cruz Biotechnology), corr4a (C4; CF Foundation modulator library), VX-809, VX-770 (both Selleck Chemicals), CFTR inhibitor-172 (inh-172; CFFT USA), forskolin (Fsk) or genistein (Gen) (both from Sigma-Aldrich). All stock solutions were made 10^3^ times concentrated, dissolved in DMSO.

### Fluorescent Iodide Influx Assay /Halide-sensitive YFP-based functional assay

CFTR activity was determined using Phe508del/HS-YFP CFBE cells seeded in 8-well chamber slides or 96-well microplates, or polarized in transwell filter inserts, as described above. Cells were treated with the indicated compound concentrations for the described time periods. Cells were washed with PBS and incubated for 30 min in PBS containing compounds for CFTR stimulation/inhibition (Fsk, VX-809, VX-770, Gen and inh172) at the indicated concentrations. Non-polarized cells were then transferred either to a microplate reader (Tecan® Infinite M200) (excitation: 505 nm and emission: 535 nm) or to a Leica TCS-SPE confocal microscope for time-lapse analysis. Each well was assayed individually for iodide influx by recording fluorescence continuously (500 ms/point) for 10 s (baseline) and then for 110 s after the rapid (<1 s) addition of isomolar PBS in which 137 mM Cl^−^ was replaced by I^−^ (PBSI, final NaI concentration in the well: 100 mM). Assays were performed at room temperature. After background subtraction, cell fluorescence recordings were normalized for the initial average value measured before addition of I^−^.

For polarized cells in transwell filter inserts, we developed an in-house setup to assess HS-YFP fluorescence decay by confocal microscopy. Briefly, a Ø 16 mm × 17 mm-high × 1mm-thick polypropylene ring was sealed on top of a glass slide to form a PBS-containing chamber (Fig. 2b), which can be positioned on the confocal microscope stage. This setup allows the transwell inserts, containing Phe508del/HS-YFP CFBE polarized cells, to be placed inside the slide chamber within the focal length of a 10× objective. The apical side medium is replaced by 100 μl PBS, and the insert secured to the chamber through a Ø 0.5 mm steel tubing clamp that also allows the addition of I^−^ and CFTR stimulators to the apical side of the polarized cell layer (Fig. 2b). XZ fluorescence images were collected continuously with a 3 Airy pinhole at 500 ms intervals, first for 10 s (baseline), and then for 110 s after the rapid (≤1 s) apical pumping of 350 μl of PBSI, containing 5 μM Fsk with or without 10 μM VX-770, and with 25 μM of inh172 for some experimental conditions. Quantification of fluorescence decay was performed on at least 30 individual cells per filter, using ImageJ (NIH) as previously described^21^.

### Immunoblotting, immunofluorescence and confocal microscopy

Samples were analysed by immunoblotting as previously described^20,21^. Additional antibodies used for WB were: mouse anti-CFTR clone 596 (obtained through the UNC CFTR antibody distribution program sponsored by CFFT), mouse anti-α-Tubulin clone B-5-1-2 (Sigma-Aldrich), mouse-anti-E-cadherin (Transduction Laboratories), mouse-anti-CK18 (Millipore), rabbit-anti-ZO-1, rabbit-anti-CK8, rabbit-anti-Ki-67 and rabbit-anti-H2B (all from Santa Cruz Biotechnology). Primary antibodies were detected using secondary, peroxidase-conjugated antibodies (Bio-Rad) followed by ECL. For densitometric analysis of WB bands, x-rays films were digitalized and images analysed with ImageJ software (NIH).

For immunofluorescence analysis, cells grown on filters were fixed with 4% formaldehyde, washed with PBS, permeabilized with 0.2% Triton X-100 (Sigma-Aldrich), and incubated for 1 h with the indicated primary antibodies. Cells were then thoroughly washed with PBS and incubated for 30 min with AlexaFluor488-conjugated secondary antibody (Life Technologies Invitrogen Corporation). Actin was stained using phalloidin-TRITC (Jackson ImmunoResearch Laboratories), followed by thorough washing in PBS and DAPI staining of nuclei. Filters were mounted on microscope slides with Vectashield (Vector Laboratories), covered with coverslips and sealed. Images were recorded on a Leica TCS-SPE confocal microscope and assembled into figures with Adobe Photoshop software.

### Statistical Analysis

Quantitative results are shown as means ± SEM of at least three independent observations. To compare sets of data, we used either ANOVA followed by Tukey’s test (for multiple treatments) or two tailed Student’s *t* tests (paired comparisons), and considered significant differences when *p* values < 0.05. In the halide-sensitive YFP-based functional assay, the signal decay caused by YFP fluorescence quenching was fitted to an exponential decay function to derive the maximal slope that corresponds to initial influx of I^−^ into the cells^21,23^. Maximal slopes were converted into rates of variation of the intracellular I^−^ concentration (in μM/s) using the equation d[I^−^]/dt = K_d_ [d(F/F_0_)/dt], where K_d_ is the affinity constant of YFP for I^−^ ^21,23^, and F/F_0_ is the ratio of the cell fluorescence at a given time versus the initial fluorescence.

## Electronic supplementary material

Supplementary Information

Supplementary Movie SM1

Supplementary Movie SM2

Supplementary Movie SM3

Supplementary Movie SM4

Supplementary Movie SM5
