# Supplementary material for: Prolonged co-treatment with HGF sustains epithelial integrity and improves pharmacological rescue of Phe508del-CFTR
Source: Sci Rep. 2018 Aug 29;8:13026. doi: 10.1038/s41598-018-31514-2 (PMC6115363; doi:10.1038/s41598-018-31514-2)
Supplement: Supplementary file 1 — Supplementary Information [file 41598_2018_31514_MOESM1_ESM.pdf]

Supplementary Information

Prolonged co-treatment with HGF sustains epithelial integrity and improves pharmacological rescue of Phe508del-CFTR

Ana M. Matos[1,2], Andreia Gomes-Duarte[1,2], Márcia Faria[1,2,3], Patrícia Barros[1,2], Peter Jordan[1,2], Margarida D. Amaral[2], and Paulo Matos[1,2,*]

Affiliations:

[1] Department of Human Genetics, National Health Institute 'Dr. Ricardo Jorge', Av. Padre Cruz, 1649-016 Lisboa, Portugal;

[2] University of Lisboa; Faculty of Sciences, BioISI – Biosystems & Integrative Sciences Institute, Campo Grande-C8, 1749-016 Lisboa; Portugal.

[3] Serviço de Endocrinologia, Diabetes e Metabolismo, do CHLN—Hospital Santa Maria, Lisboa, Portugal

[*] Address correspondence to: Dr. Paulo Matos, BioISI – Biosystems & Integrative Sciences Institute, Faculty of Sciences, University of Lisboa, Campo Grande-C8 1749-016 Lisboa, Portugal; phone: +351-217 500 000; e-mail: phmatos@fc.ul.pt

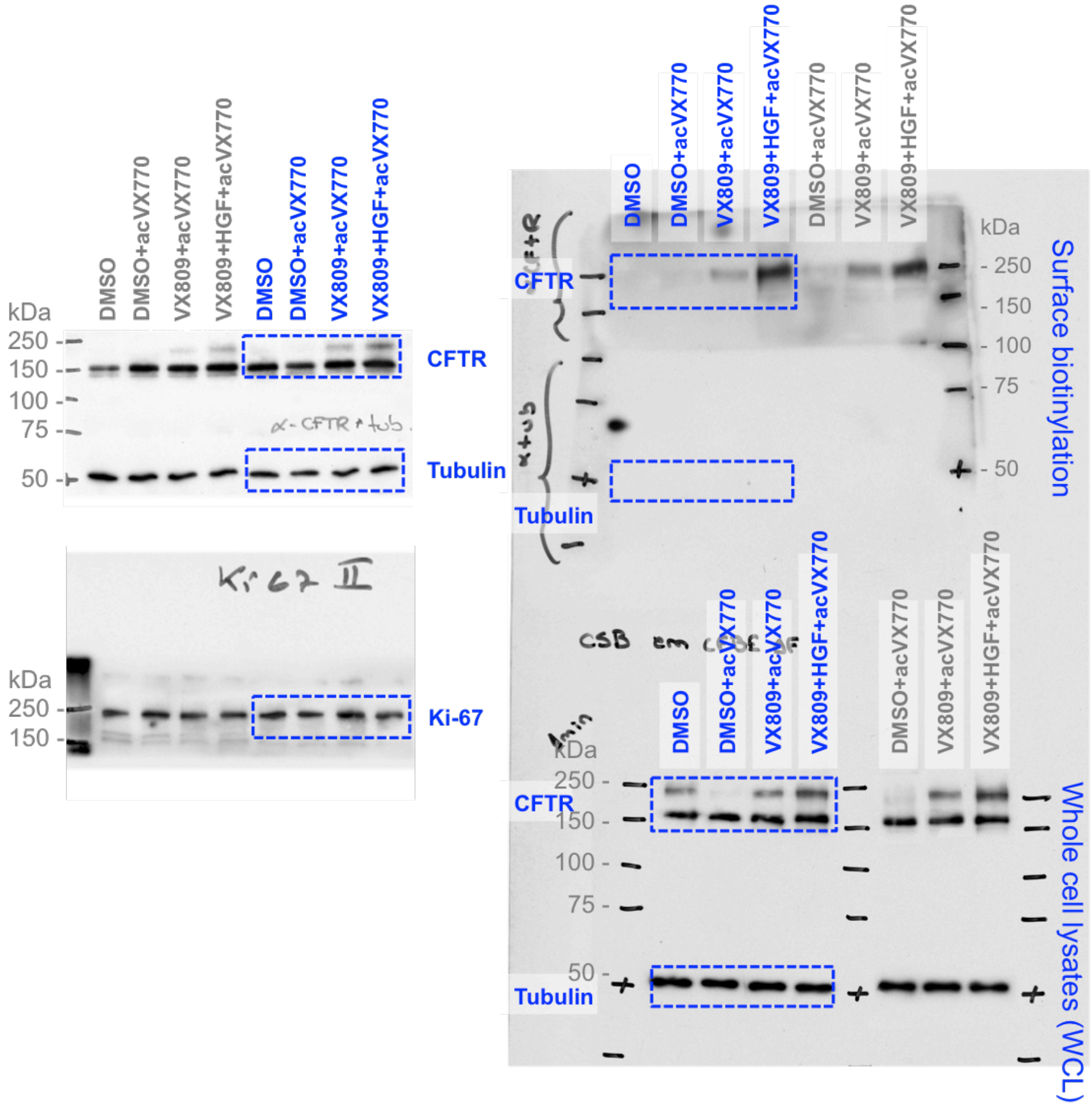

Supplementary Figure S1: Full-length Western blots used to assemble Figures 3a (left) and 3e (right). Blue lettering indicates sample and antibody information included in the Figures and dotted line rectangles indicate the cropped blot regions used for the Figure panels. Grey lettering indicates additional information on the blots that was not included in the Figures.

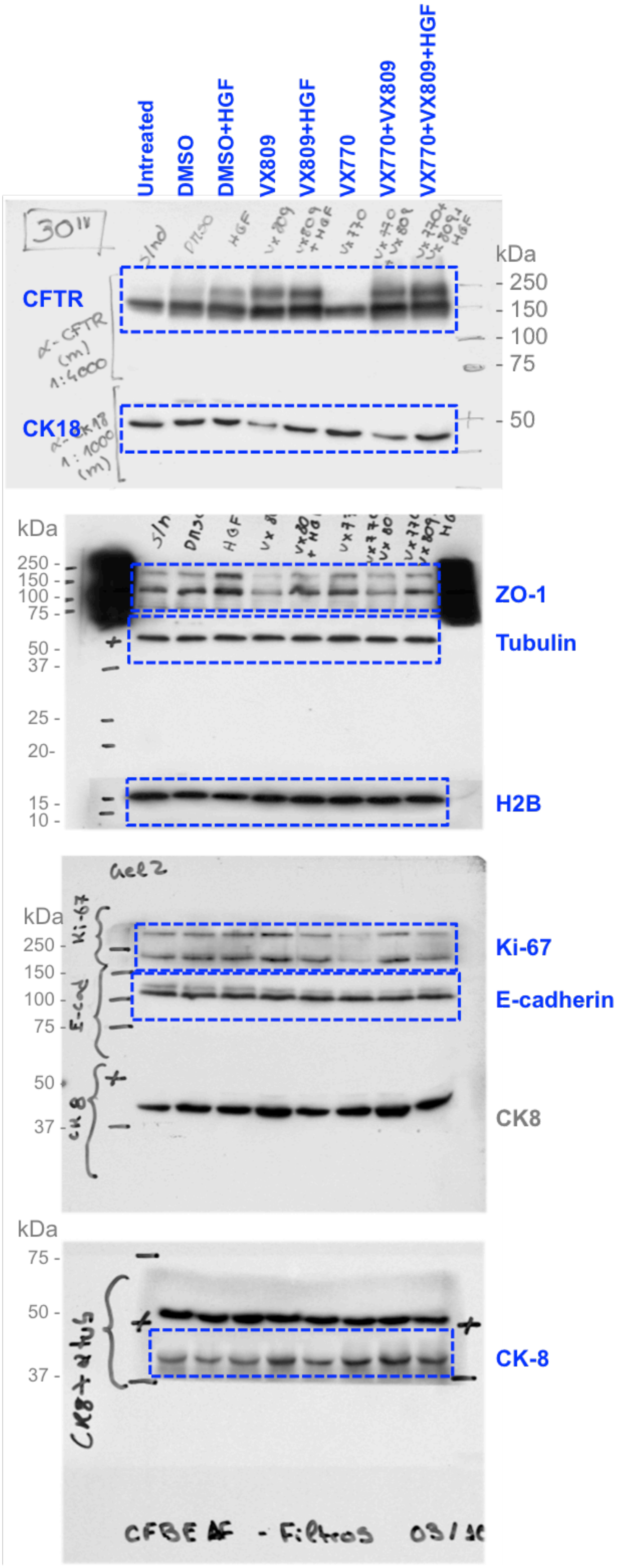

Supplementary Figure S2: Full-length Western blots used to assemble Figures 4b and 5a. Blue lettering indicates sample and antibody information included in Figures 4b and 5a. Note that sample order is the same in all blots. Dotted line rectangles indicate the cropped blot regions used for the panels shown in the Figures. Grey lettering indicates additional information on the blots that was not included in the Figures.

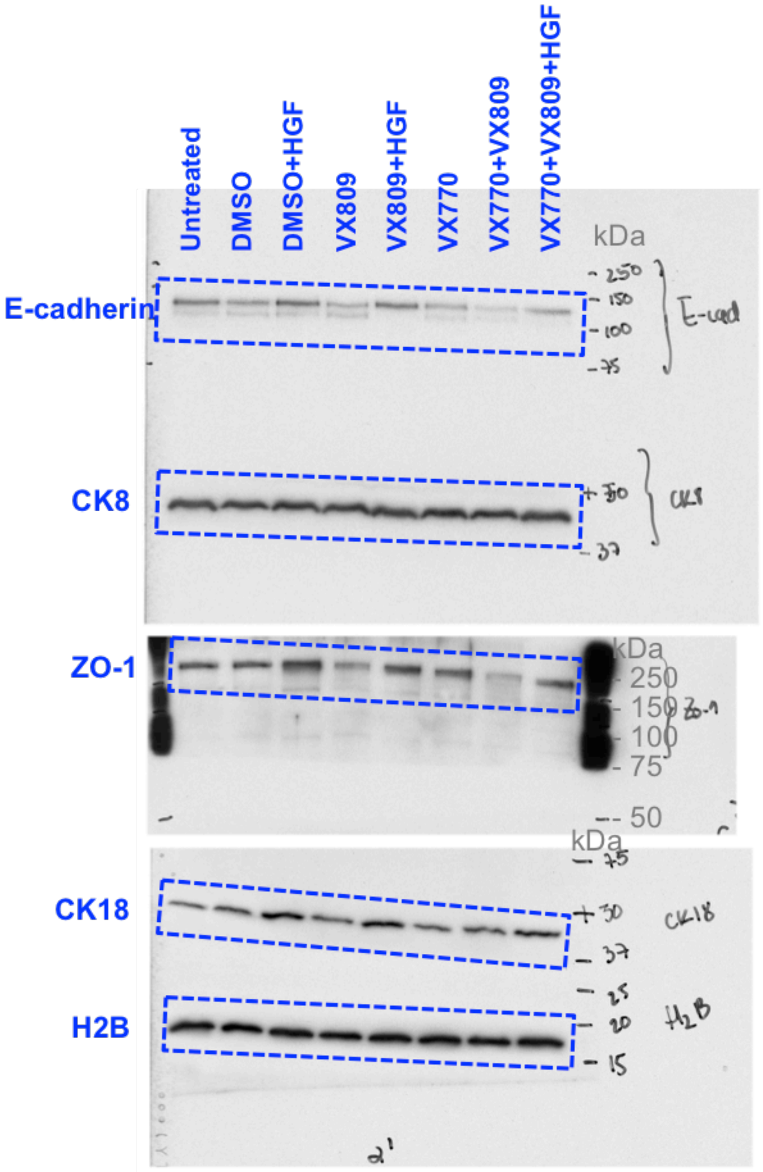

Supplementary Figure S3: **Full-length Western blots used to assemble Figure 6c**. Blue lettering indicates sample and antibody information included in Figure 6c. Note that sample order is the same in all blots. Dotted line rectangles indicate the cropped blot regions used for the panels shown in the Figure. Grey lettering indicates additional information on the blots that was not included in the Figure.

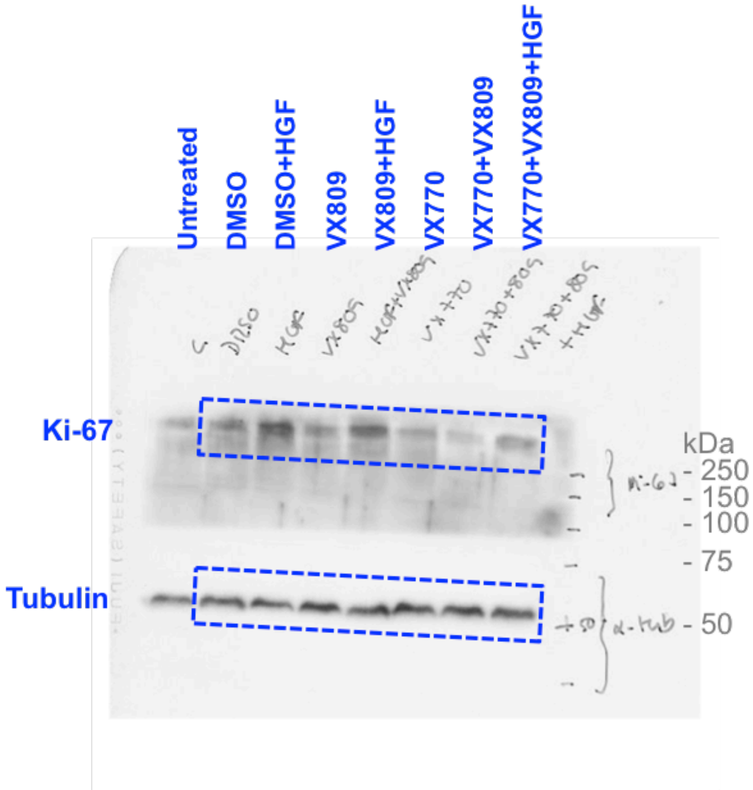

Supplementary Figure S4: **Full-length Western blots used to assemble Figure 7**. Blue lettering indicates sample and antibody information included in Figure 7. Dotted line rectangles indicate the cropped blot regions used for the panels shown in the Figure. Grey lettering indicates additional information on the blots that was not included in the Figure.

Supplementary Movie SM1: Representative recording of YFP quenching by iodide influx, in polarized CFBE-Phe508del/HS-YFP cells treated for 48 h with DMSO. Shown is a sequence of XZ confocal fluorescence images, collected continuously after the apical addition of iodide (indicated as "+Iodide") together with 5 µM Forskolin. Time stamp is shown in the upper right corner and the white bar in the bottom left corner represents 50 µm.

Supplementary Movie SM2: Representative recording of YFP quenching by iodide influx, in polarized CFBE-Phe508del/HS-YFP cells treated for 48 h with DMSO. Shown is a sequence of XZ confocal fluorescence images, collected continuously after the apical addition of iodide (indicated as "+Iodide") together with 5 µM Forskolin and 10 µM of VX-770. Time stamp is shown in the upper right corner and the white bar in the bottom left corner represents 50 µm.

Supplementary Movie SM3: Representative recording of YFP quenching by iodide influx, in polarized CFBE-Phe508del/HS-YFP cells treated for 48 h with 3 µM of VX-809. Shown is a sequence of XZ confocal fluorescence images, collected continuously after the apical addition of iodide (indicated as "+Iodide") together with 5 µM Forskolin and 10 µM of VX-770. Time stamp is shown in the upper right corner and the white bar in the bottom left corner represents 50 µm.

Supplementary Movie SM4: Representative recording of YFP quenching by iodide influx, in polarized CFBE-Phe508del/HS-YFP cells treated for 48 h with 3 µM of VX-809 and with 50 mg/ml HGF for the last 24 h. Shown is a sequence of XZ confocal fluorescence images, collected continuously after the apical addition of iodide (indicated as "+Iodide") together with 5 µM Forskolin and 10 µM of VX-770. Time stamp is shown in the upper right corner and the white bar in the bottom left corner represents 50 µm.

Supplementary Movie SM5: Representative recording of YFP quenching by iodide influx, in polarized CFBE-Phe508del/HS-YFP cells treated for 48 h with 3 µM of VX-809 and with 50 mg/ml HGF for the last 24 h. Cells were then pre-treated for 15 min with 25 µM of inh172, prior to CFTR stimulation. Shown is a sequence of XZ confocal fluorescence images, collected continuously after the apical addition of iodide (indicated as "+Iodide") together with 5 µM Forskolin, 10 µM of VX-770 and 25 µM of inh172. Time stamp is shown in the upper right corner and the white bar in the bottom left corner represents 50 µm.
